# Supplementary material for: Identification and functional analysis of non-coding regulatory small RNA FenSr3 in Bacillus amyloliquefaciens LPB-18
Source: PeerJ. 2023 May 15;11:e15236. doi: 10.7717/peerj.15236 (PMC10194069; doi:10.7717/peerj.15236)
Supplement: Supplemental Information 4 [file peerj-11-15236-s004.zip › KO/CK-vs-T1_map/map04012.html]

KEGG PATHWAY: ErbB signaling pathway - Reference pathway


|  |  |
| --- | --- |
| **ErbB signaling pathway - Reference pathway** |  |

[
Pathway menu
| Organism menu
| Pathway entry
| Show description
| User data mapping
]

The ErbB family of receptor tyrosine kinases (RTKs) couples binding of extracellular growth factor ligands to intracellular signaling pathways regulating diverse biologic responses, including proliferation, differentiation, cell motility, and survival. Ligand binding to the four closely related members of this RTK family -epidermal growth factor receptor (EGFR, also known as ErbB-1 or HER1), ErbB-2 (HER2), ErbB-3 (HER3), and ErbB-4 (HER4)-induces the formation of receptor homo- and heterodimers and the activation of the intrinsic kinase domain, resulting in phosphorylation on specific tyrosine residues (pY) within the cytoplasmic tail. Signaling effectors containing binding pockets for pY-containing peptides are recruited to activated receptors and induce the various signaling pathways. The Shc- and/or Grb2-activated mitogen-activated protein kinase (MAPK) pathway is a common target downstream of all ErbB receptors. Similarly, the phosphatidylinositol-3-kinase (PI-3K) pathway is directly or indirectly activated by most ErbBs. Several cytoplasmic docking proteins appear to be recruited by specific ErbB receptors and less exploited by others. These include the adaptors Crk, Nck, the phospholipase C gamma (PLCgamma), the intracellular tyrosine kinase Src, or the Cbl E3 ubiquitin protein ligase.

|  |  |  |
| --- | --- | --- |
| Reference pathway | 184% 150% 122% 100% 82% 67% 55% | 图片下载 |
